# Application of an ultrasonic bone knife combined with a dental electric motor in the extraction of mandibular middle and low impacted teeth

**DOI:** 10.1186/s12903-023-03788-0

**Published:** 2024-01-04

**Authors:** Qian Wang, Tonghan Zhang

**Affiliations:** 1 School of Stomatology, Jinan University, Guangzhou, 510000 China; 2 Department of Oral and Maxillofacial Surgery, Hospital of Stomatology, Zhongshan City, Zhongshan, 528400 China

**Keywords:** UBK, DEM, turbo drilling method, BCSC, Mandibular middle and low impacted teeth

## Abstract

**Objective:**

To investigate the clinical application of an ultrasonic bone knife (UBK) combined with a dental electric motor (DEM) in the extraction of mandibular middle and low impacted teeth.

**Methods:**

From January 2022 to May 2023,200 patients with wisdom teeth were randomly divided into three groups: experimental group A (UBK combined with DEM), experimental group B (UBK combined with high-speed turbine mobile phone (HSTMP)), and the control group (bone chisel split crown (BCSC)). The operation time, psychological state during operation, pain, swelling, limitation of mouth opening and other complications on the first, second and third days after operation were recorded.

**Results:**

The operation time of experimental group A (EAG) (12.95 ± 2.12) minutes was shorter than that of experimental group B (EBG) (17.06 ± 2.25) minutes and the control group (CG) (23.43 ± 2.18) minutes, and the difference was statistically significant (*P* < 0.05). The psychological state of the EAG was significantly lower than that of the EBG and CG (*P* < 0.05). The postoperative pain, swelling, limitation of mouth opening and complications in the EAG were significantly lower than those in the EBG and CG (*P* < 0.05).

**Conclusion:**

UBK combined with DEM in the extraction of mandibular middle and low obstructed teeth has good results, good prognosis, high safety, short operation time, better psychological status of patients, low postoperative pain, swelling, mouth opening restriction and complication rate, and is currently the preferred extraction method.

**Supplementary Information:**

The online version contains supplementary material available at 10.1186/s12903-023-03788-0.

## Introduction

With the development and progress of society, the influence of multiple factors, such as racial evolution, bad habits and chewing food structure, causes the jaw bone length to be incongruent with the size of the teeth, making it easy to form interceptive teeth. They generally grow slowly and are mostly seen in adolescents, but there is great variability. Middle and low impacted wisdom teeth in the lower jaw are more dangerous. For example, first, they easily cause food impaction and pericoronitis; second, they may squeeze the neighboring teeth and cause caries or even fall off; third, they cause distal mesial bone resorption or distal mesial root resorption of neighboring teeth; fourth, they cause malocclusion and serious occlusal disorder, which causes temporomandibular joint disorder; fifth, they cause systemic infection and threaten life.

The classification of impacted teeth can be divided into: 1) high impacted teeth: the highest part of the impacted teeth crown is parallel to or slightly above the plane of the entire dental arch; 2) median impacted teeth: the highest position of the teeth is higher than the neck of the second molar, but lower than the position of the (occlusal) plane; 3) low impacted teeth: it means that the highest position of the crown of the impacted teeth is lower than the neck of the second molar [1].

Cone beam computed tomography (CBCT) has been widely applied in the field of dentistry because of its high imaging resolution, relatively low radiation dose, and ability to perform three-dimensional reconstruction imaging [2, 3]. However, the methods for surgical extraction of mandibular medium and low obstructed wisdom teeth are minimally invasive procedures such as UBK combined with DEM and ultrasonic osteotome combined with turbine handpiece; and conventional procedures such as BCSC, amputation method, orthodontic traction-assisted method and peri-coronal debridement method.

The UBK is a new type of minimally invasive extraction procedure; this device uses ultrasonic frequencies to cut bone with micro vibrations between 60 and 200 mm/s and a frequency range of 24-29 kHz [4, 5]. These micro-vibrations cut accurately and avoid damage to soft tissues and are an important embodiment of the minimally invasive concept in oral and maxillofacial surgery clinics by virtue of their characteristic safety, precision, comfort, and speed and time savings [6, 7]. Some studies have shown that UBK has the function of accurate identification and selective cutting. In the process of bone removal or gap increase, it will immediately stop working when adjacent to important anatomical structures such as nerves and blood vessels and effectively protect soft tissues. The efficiency of cutting tooth tissue is low, which prolongs the operation time and aggravates the fear and tension of patients during tooth extraction [8].

In the past, the widely used high-speed turbine handpiece can produce a large amount of noise during the operation, and the cutting force, torque, specific cutting energy and burr wear increase accordingly due to the increase in its material removal rate, while the rotational speed decelerates until it stops, which greatly increases the patient’s fear and the operation time [9]; its high-speed cutting easily damages the surrounding soft tissues and causes subcutaneous emphysema, and easily damages nerves and blood vessels. With the advent of dental electric motors, the problems of low efficiency of high-speed turbine handpieces in cutting dental tissue and subcutaneous emphysema caused by jet airflow into the tissue have been solved [10, 11]. The bone chisel crown technique is highly sensitive, not easy to master, and has high postoperative complications, such as dry socket, it is one of the complications after tooth extraction and usually occurs in the mandibular molars. Severe pain 2 to 3 days after surgery, painkillers do not relieve pain, and the extraction socket is empty and sometimes has a rancid smell [12]. But its technique is economical.

The new DEM includes a housing, a rotor, an end cap, a water pipe, a gas pipe, a lamp cord and a motor stator laminate; four through-holes are provided in the motor stator laminate to form the water pipe and gas pipe passage through-holes and two circuit passage through-holes. When the dental drill shell diameter remains unchanged, the overall space of the dental electric motor is effectively utilized, and its rotational speed and working power are substantially increased. Moreover, when subjected to excessive pressure, its rotational speed decay is greatly improved, and it basically maintains a constant speed to cut dental hard tissues, which greatly reduces the operation time; at the same time, the rate of temperature increase is greatly improved during long time and overload work, the service life of the electric motor is prolonged, and failure is reduced. The DEM provides auxiliary light illumination; in the treatment of maxillary teeth, posterior teeth, broken roots and other parts, high-speed turbine handpieces often have the problem of insufficient light, prolonging the operation time and reducing patient satisfaction.

Based on the known advantages of UBK and DEM, we assume that the operation can relieve pain, swelling, and limited opening and reduce the incidence of postoperative complications after mandibular third molar extraction. Therefore, this paper compares the psychological state, operation time, postoperative pain, swelling, degree of mouth opening, nerve injury and joint discomfort of three groups by analyzing patients in the EAG, EBG and CG.

## Materials and methods

### General information

From January 2022 to May 2023,200 patients (200 teeth) with mandibular middle and low impacted wisdom teeth were selected from the Department of Oral and Maxillofacial Surgery, Zhongshan People’s Hospital. They were randomly divided into three groups: in EAG, UBK (Settler KJF800) combined with COXO DEM C-PUMA) 84 cases; in EBG, there were 53 cases of UBK combined with HSTMP; and in EBG, 63 cases were chiseled. There were 74 males and 126 females, with an average age of (26.97 ± 6.05) years. There was no significant difference in sex or age among the three groups (*P* > 0.05). See Table 1. The study design and methods were approved by the Ethics Committee of Zhongshan People’s Hospital, Ethics No. 2023-018.

**Table 1:** Comparison of general information of the three groups of patients

Group	Number of cases	Male/female (cases)	Age (years)
EAG	84	30/54	26.49 ± 6.72
EBG	53	22/31	27.79 ± 4.46
ECG	63	22/41	26.92 ± 6.26
χ^2^ / F		0.64	0.76
*P*		0.73	0.47

### Research objects

Inclusion criteria: patients diagnosed with mandibular middle and low impacted wisdom teeth; patients were aware of and agreed to this procedure; good physical condition, no history of allergy or radiation or chemotherapy, capable of surgical treatment; presence of adjacent teeth, coronal tissue and root resistance; good compliance and timely follow-up.

Exclusion criteria: patients with unsound relevant data; unexplained withdrawal from activities during the investigation; restricted mouth opening; women during pregnancy, lactation or menstruation; and patients with acute inflammation or systemic diseases.

### Surgical methods

#### Preoperative preparation

CBCT was used to further examine the positional relationship between the root and the mandibular canal and further analyze the resistance. All patients were given metronidazole (0.4 g, 2 times/d), cefuroxime tablets (0.5 g, 2 times/d), dexamethasone (1.5 mg, 2 times/d), and ibuprofen sustained-release capsules (0.3 g, 2 times/d) for 3 days before the operation, while avoiding taking other types of antibiotics at the same time. First, the mouth was rinsed with mouthwash and disinfected around the mouth. Inferior alveolar nerve block anesthesia and peri-coronal local infiltration were performed with 1.8 mL (Ziphodont, France) of 3% mepivacaine hydrochloride. Local submucosal infiltration was used for infiltration anesthesia to achieve hemostasis and analgesia. Conventional disinfection towel.

#### Surgical methods

**Fig. 1:**
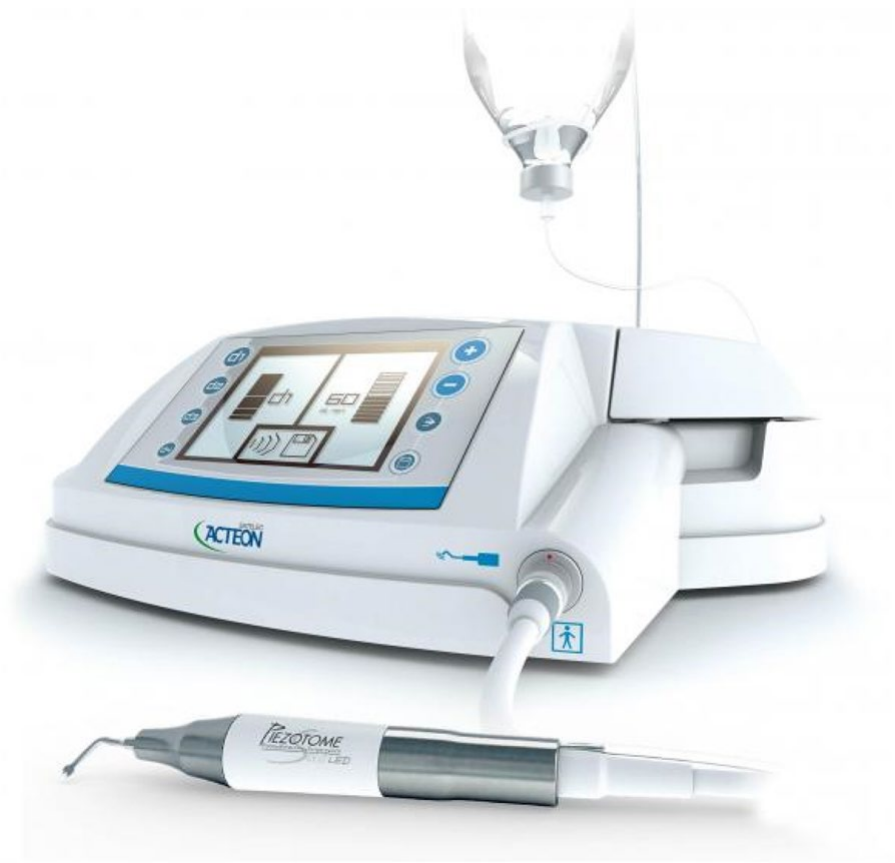
UBK

**Fig. 2:**
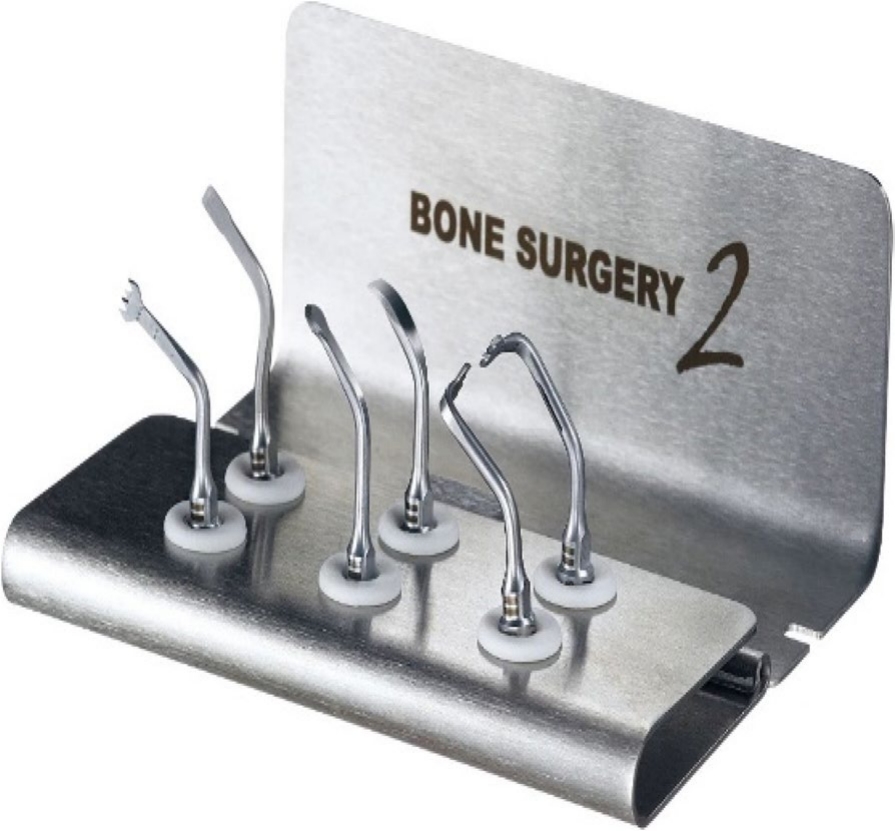
Work Tip

**Fig. 3:**
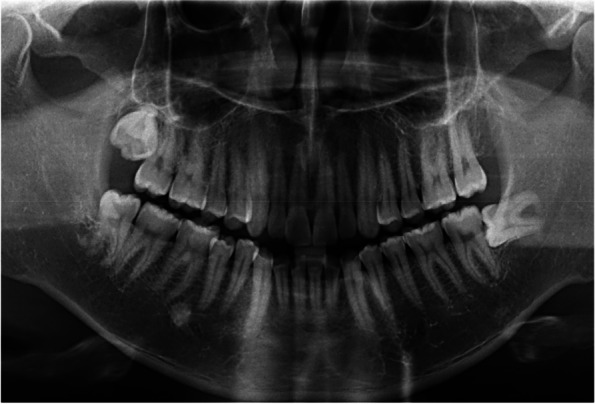
Preoperative panoramic film

**Fig. 4:**
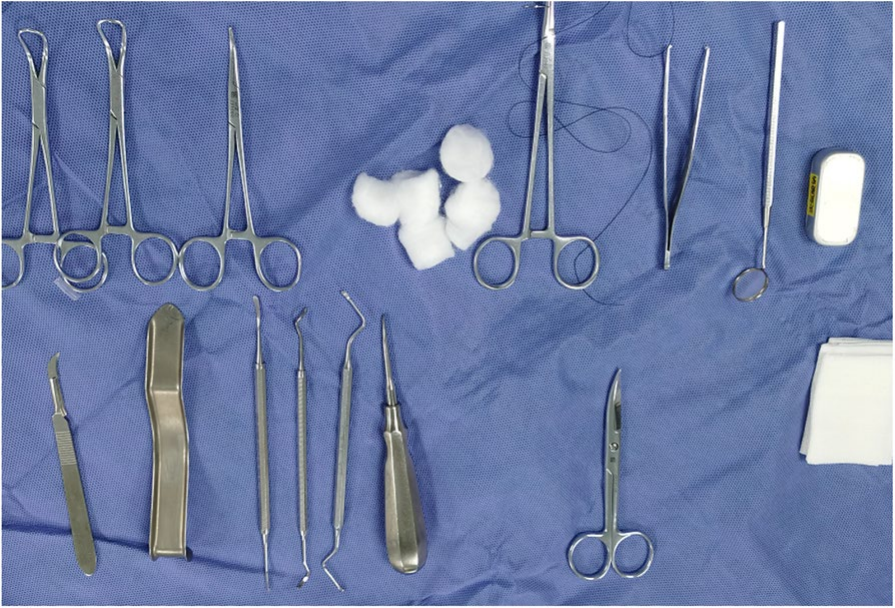
Preoperative surgical instruments

**Fig. 5:**
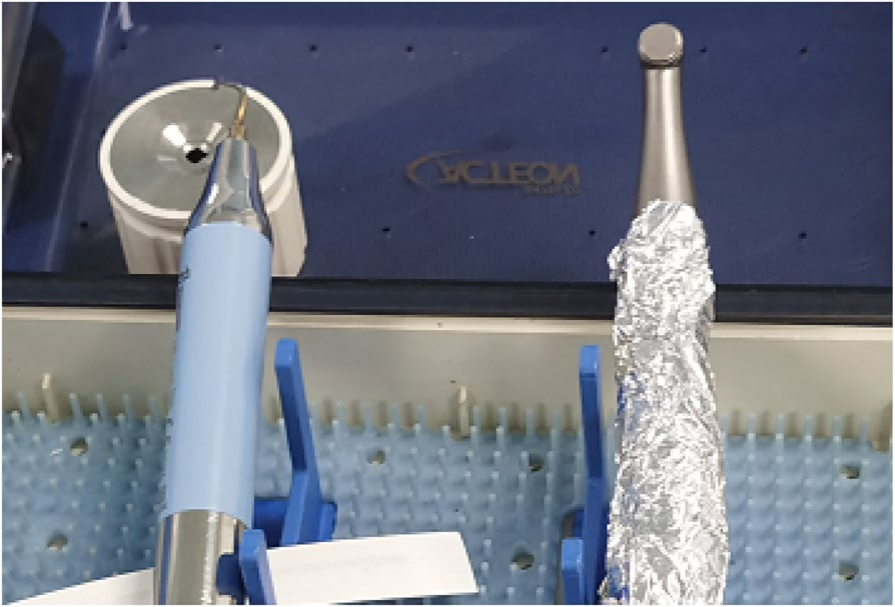
UBK handle and EDM

**Fig. 6:**
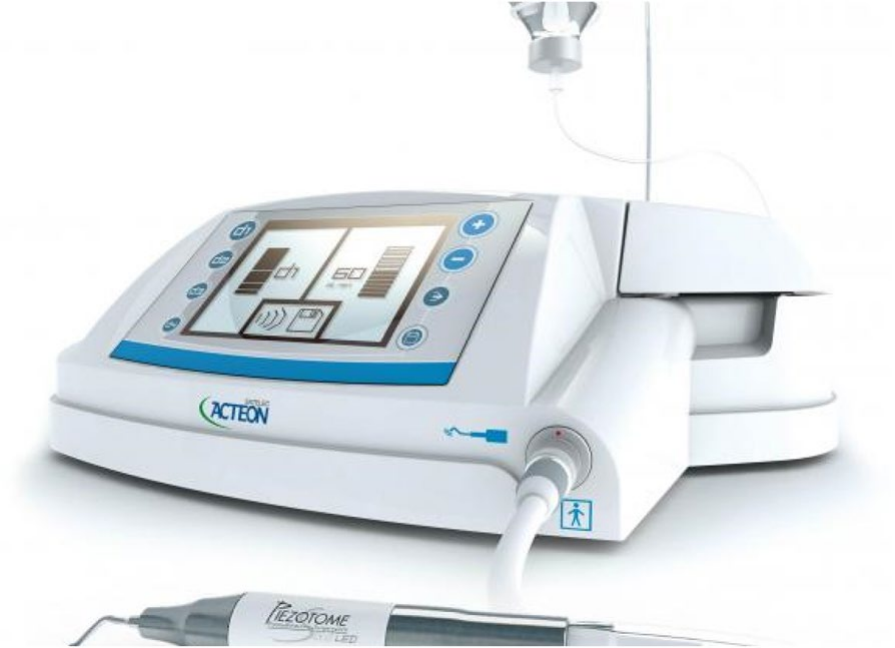
Settler UBK

**Fig. 7:**
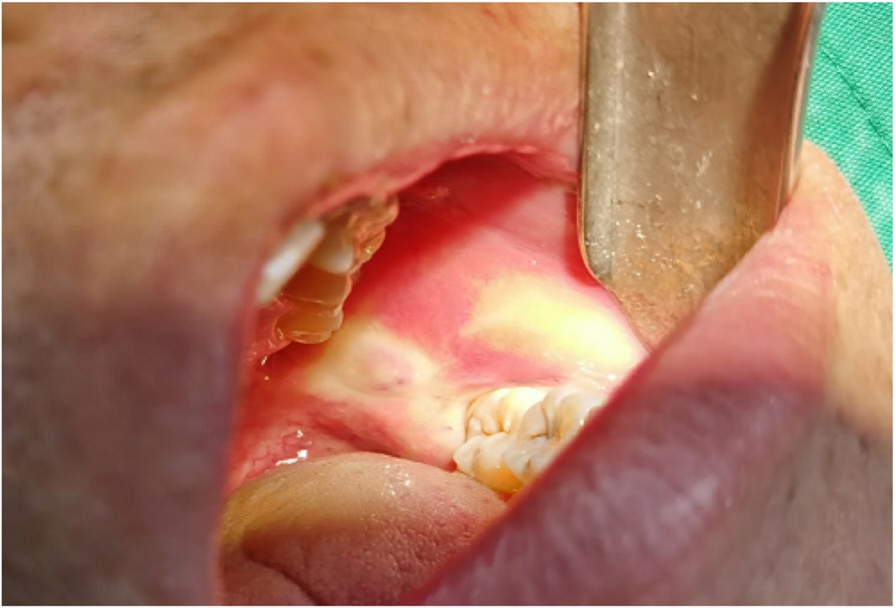
Intraoral

**Fig. 8:**
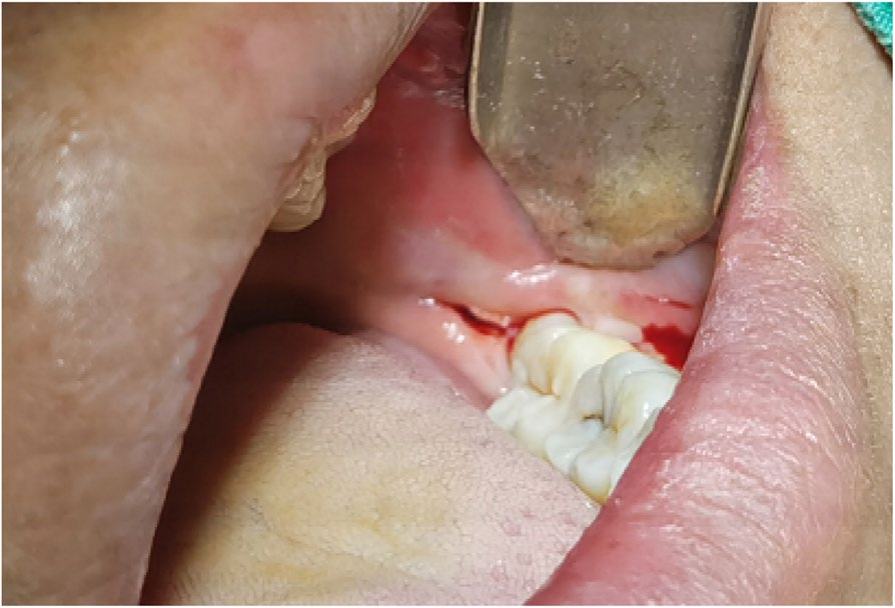
Cutting

**Fig. 9:**
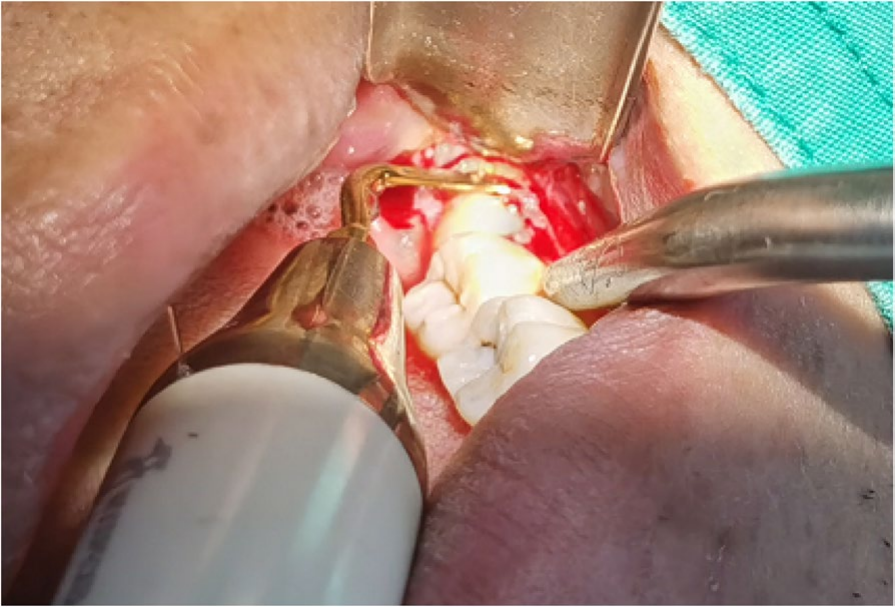
Ultrasonic bone debridement and gap augmentation

**Fig. 10:**
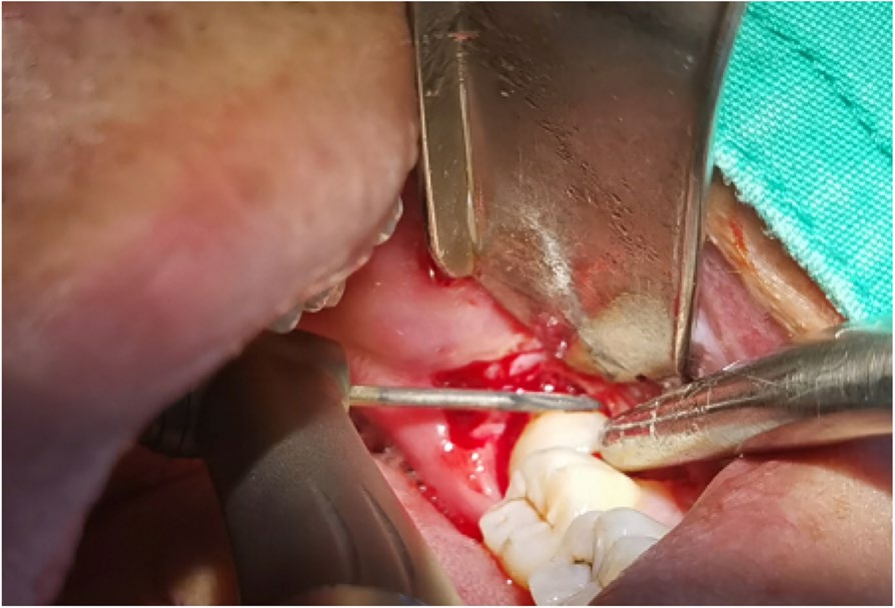
Electric motor “T” split teeth

**Fig. 11:**
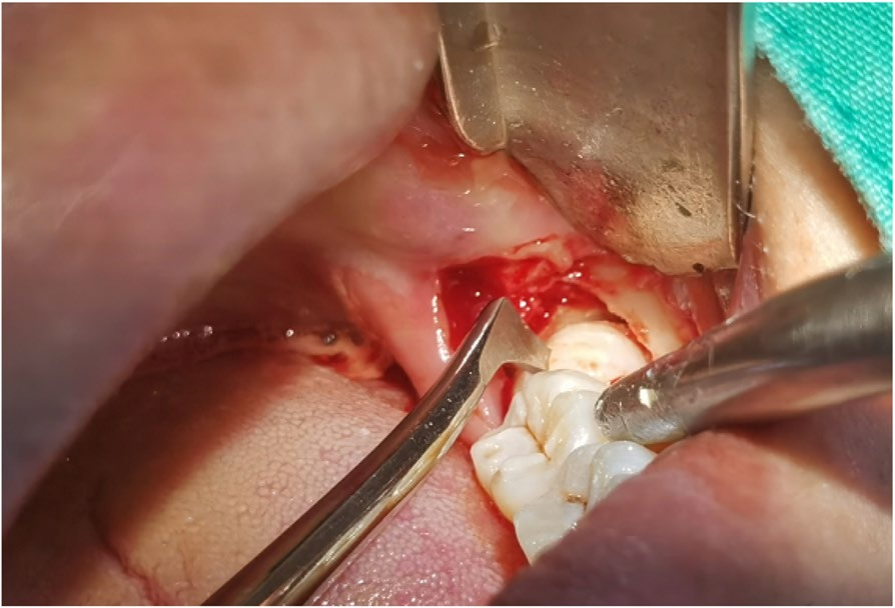
The crown of a loose tooth

**Fig. 12:**
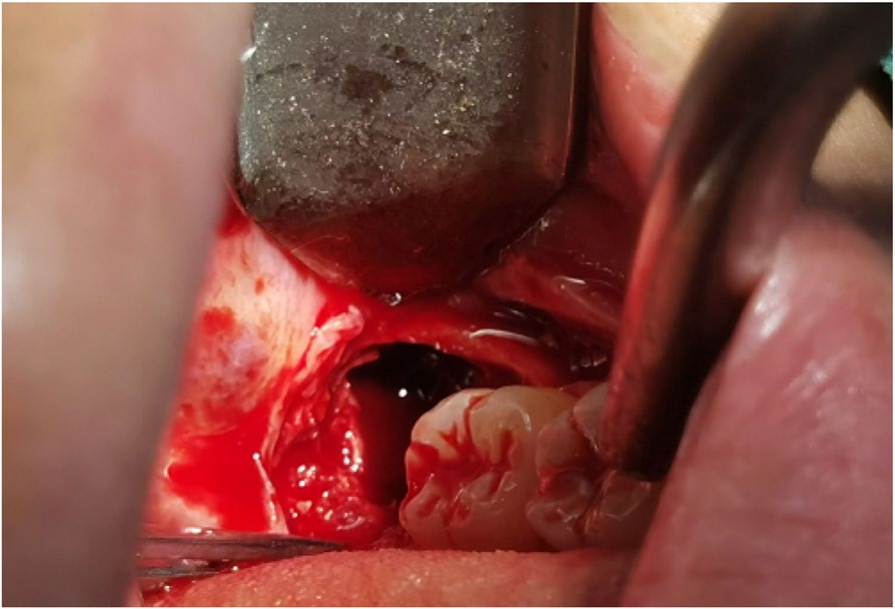
Removal of crown and root

**Fig. 13:**
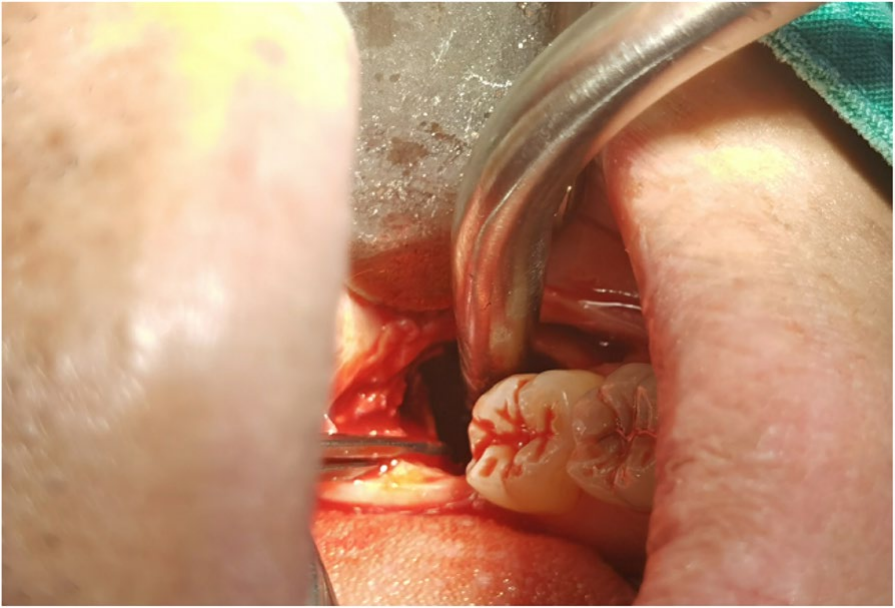
Scraping and flushing of extraction sockets

**Fig. 14:**
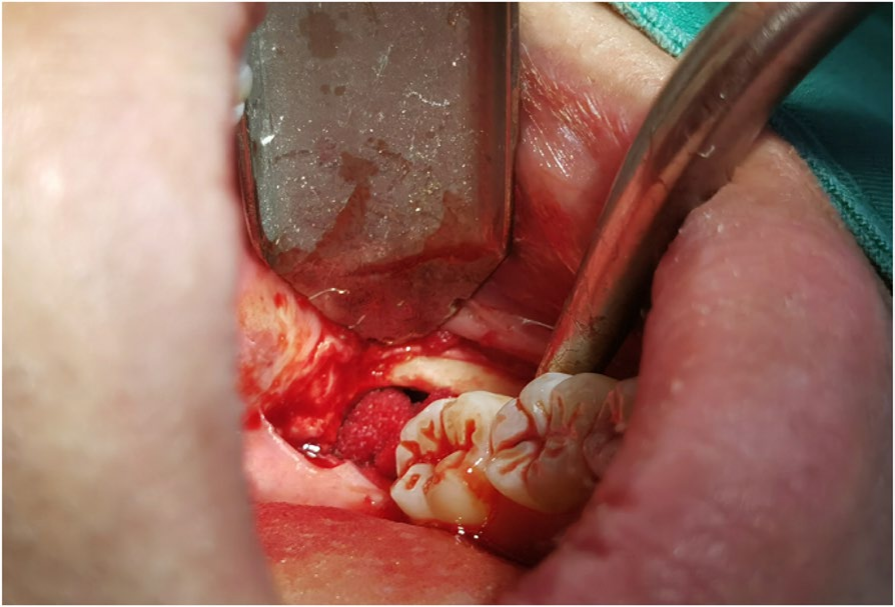
Put in collagen sponge

**Fig. 15:**
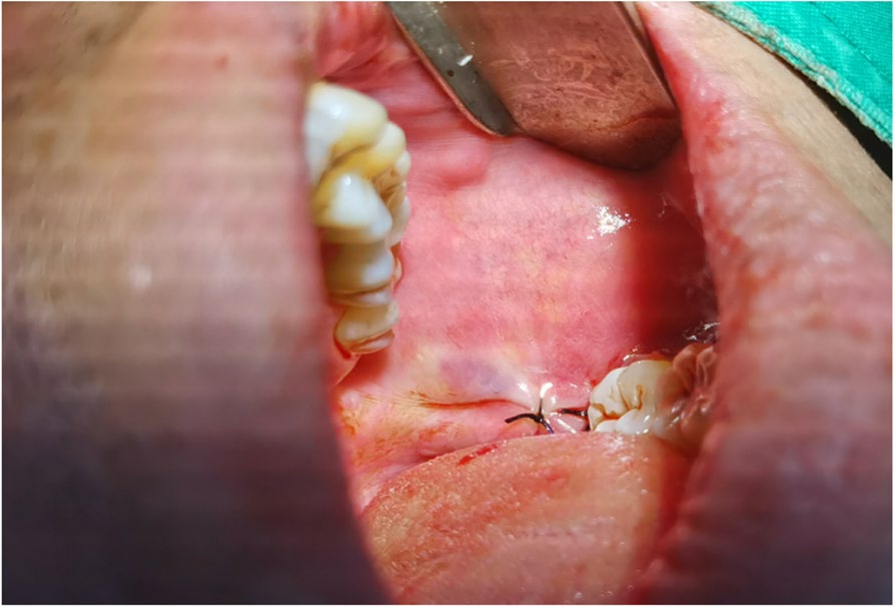
Suture

**Fig. 16:**
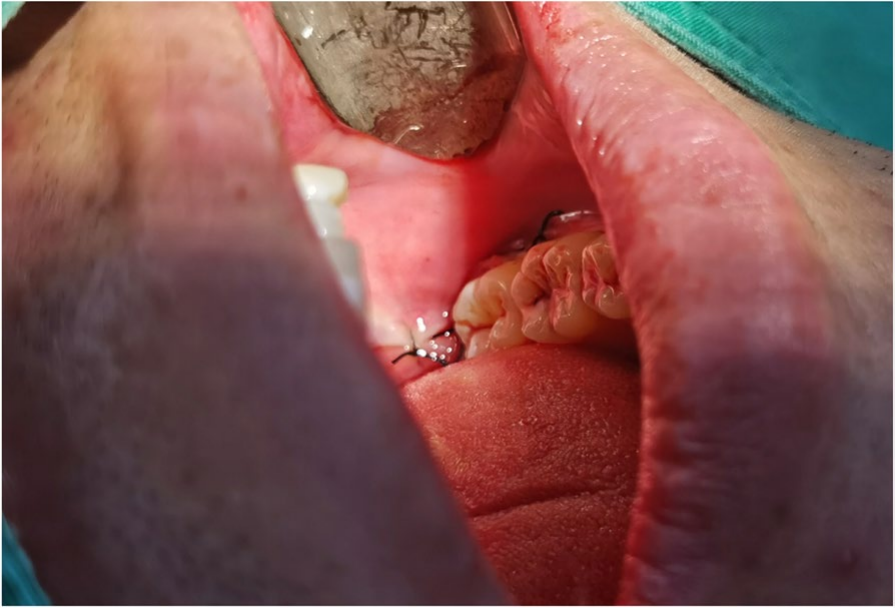
Postoperative intraoral

**Fig. 17:**
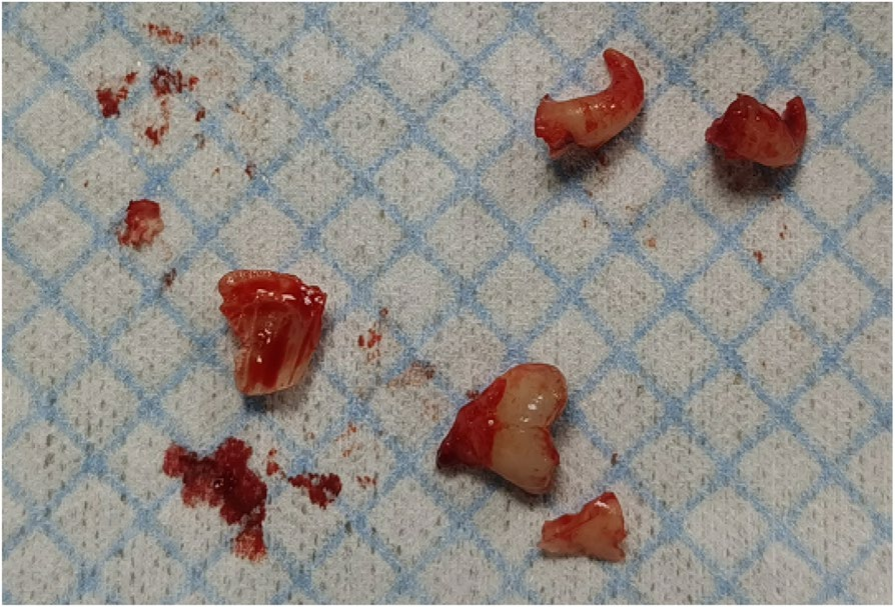
38 teeth

**Fig. 18:**
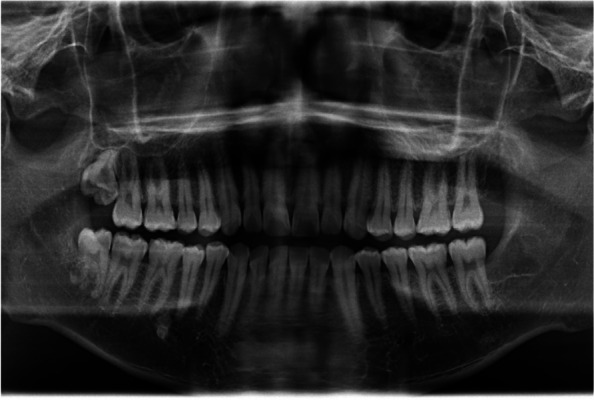
Postoperative panoramic film

1. In EAG, the operation was as follows: the triangular flap was designed, the buccal side of the distal midline of the second molar was incised, and the additional incision was located in the mesial quarter of the second molar, which was 45° to the gingival margin. The UBK is set to the cortical bone cutting mode. The UBK (US1 knife head) (See Figs. 1 and 2) removes the bone around the mandibular third molar, exposes the maximum circumference of the third molar, and uses the DEM to cut the crown and divide the teeth. After removing the tooth resistance, the crown is removed, and the UBK (UC1 or UC5) (see Fig. 2) is used to increase the gap around the root to create a fulcrum. The root is removed with the teeth, the dental follicle or granulation tissue is cleaned, the residual bone fragments or tooth fragments are removed, the irregular bone tip is trimmed, the alveolar fossa is rinsed with normal saline, the alveolar fossa is soaked with gentamicin for 1-2 minutes, 10 mg dexamethasone is injected around the extraction socket, and the soft tissue in place is sutured. Compression hemostasis. Observation of postoperative intraoral conditions and retaping. See Figs. 3, 4, 5, 6, 7, 8, 9, 10, 11, 12, 13, 14, 15, 16, 17 and 18.

2) In EBG, similar to EAG, a high-speed turbine handpiece was used.
3) In the CG, the traditional chiseling and splitting crown method was used for treatment. The operation was as follows: design triangular flap, cut the distal midline of the second molar to the buccal side, and the additional incision was located in the mesial quarter of the second molar obliquely to the mesial side, 45° with the gingival margin, turn the mucoperiosteal flap, expose the bone surface and stop bleeding in time. The round bone chiseled a vertical incision on the distal bone surface of the second molar, cut off the stress, avoid damaging the bone plate on the buccal side of the second molar, chisel off the distal bone tissue of the second molar, and fully expose the crown to the maximum circumference as far as possible. According to the root of the tooth, it is decided whether to split the mesial crown or the median crown. Protruding broken tooth tissue, cleaning up dental sac or granulation tissue, trimming irregular bone tips to remove residual bone fragments or tooth fragments, suturing soft tissue in the opposite position, and compressing hemostasis.

### Evaluation indicators

#### Operation time: the time from gingival incision to the completion of wound suture

##### Psychological state

The Modified Dental Anxiety Scale (MDAS) [13] was used to evaluate the anxiety of patients during operation. The scale includes 4 items. Each was 1-5 points, with a full score of 20 points. The higher the score, the more obvious the anxiety of patients. Level 1 indicates very nervous (MDAS score > 16-20), level 2 indicates nervous (MDAS score > 13-16), level 3 indicates somewhat nervous (MDAS score > 8-13), and level 4 indicates no nervousness (MDAS score 4-8).

##### Postoperative complications

The quantitative scores of pain on the 1st, 2nd and 3rd days after the operation, facial swelling on the 1st day after the operation and limitation of mouth opening on the 1st day after the operation were recorded.

- ① Pain: The visual analog score (VAS) was used to quantify [14] the degree of postoperative wound pain, A VAS value of 0-10, where 0 indicates complete painlessness and 10 indicates unbearable pain, and the larger the number is, the more severe the pain.
- ② Limitation of mouth opening: The vertical distance between the upper and lower central incisors was measured to quantify the degree of opening limitation. no mouth opening restriction: > 2.5 cm, rated as 0; mild: 2.0-2.5 cm, rated as 1; moderate: 1.0-2.0 cm, rated as 2; severe: < 1.0 cm, rated as 3; complete mouth opening restriction: tightly closed teeth, rated as 4.
- ③ Facial swelling: The Alcantara method was used to quantify the degree of facial swelling, and the preoperative and postoperative affected corners of the mouth B (B′), the anterior ear screen A (A’), the external canthus C (C′), and the mandibular angle (D′) were selected as reference points to measure the preoperative and postoperative AB (A ‘B’), CD(C’D’) the sum of body surface distances, and the difference ((L) indicates the degree of swelling [15]. Basically normal appearance (0): L < 3 mm.

**Fig. 19:**
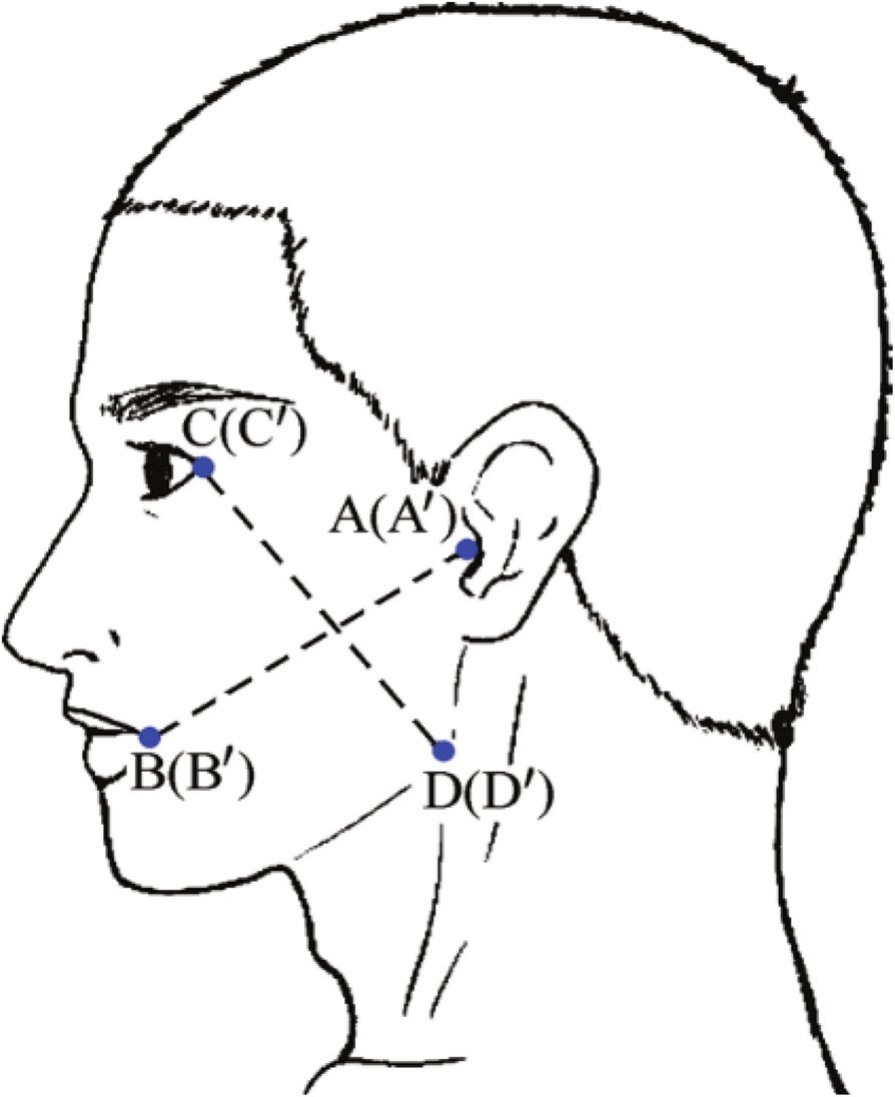
Schematic diagram of postoperative swelling [15]

Mild swelling (1): 3 mm < L < 8 mm; moderate swelling (2): 8 mm < L < 13 mm; severe swelling (3): L > 13 mm (as in Fig. 19).

The number of cases of postoperative complications, such as Wound infection (WI), dry socket disease (DSD), lower lip numbness (LLN), facial bruising (FB) (Subcutaneous emphysema) and joint discomfort (JD), were recorded, analyzed and compared.

### Statistical analysis

SPSS 26.0 software package was used for statistical analysis. Count data were expressed as the composition ratio or rate (%), and the chi-square test was used for comparisons between groups. The measurement data conforming to the normal distribution were expressed as the mean ± standard deviation ($\overline{x}$ ± s). Analysis of variance was used for comparison among multiple groups, and the least significant difference-t (LSD-t) test was used for further pairwise comparison. *P* < 0.05 was considered statistically significant.

## Results

### Comparison of operation time among the three groups

All patients successfully completed the operation at one time. The operation times of the EAG, EBG and CG were 12.95 ± 2.11 minutes, 17.06 ± 2.25 minutes and 23.43 ± 2.18 minutes, respectively. The difference was statistically significant (*P* < 0.05). The operation time of the EAG was shorter than that of the EBG and CG. See Table 2.

**Table 2:** Surgical time of patients in the three groups (*n* = 200, $\overline{x}$ ± s)

Group	Surgery time (minutes)
EAG	12.95 ± 2.11
EBG	17.06 ± 2.25
CG	23.43 ± 2.18
χ [2] /F	420.67
*P*	0.00

### Comparison of the degree of psychological state in the three groups of patients

There was a significant difference in the degree of psychological state among the three groups (H = 65.376, *P* < 0.05), and EAG was better than EBG and CG. See Table 3.

**Table 3:** Comparison of the degree of psychological state in three groups of patients during operation (n = 200, cases (%))

Group	Number of cases	1分	2分	3分	4分
EAG	84	5(6%)	13(15.5%)	36(42.9%)	30(35.7%)
EBG	53	3(5.7%)	10(18.9%)	22(41.5%)	18(34%)
CG	63	12(19%)	26(41.3%)	16(25.4%)	9(14.3%)
H	65.376				
*P*	0.000				

### Comparison of VAS values of pain on the first, second and third day after operation among the three groups of patients

On the first day after the operation, the VAS values of pain in the three groups were 3.89 ± 0.96,4.19 ± 1.29 and 4.92 ± 1.05, respectively, with a significant difference (*P* < 0.05). On the second day after the operation, the VAS values of pain in the three groups were 3.05 ± 1.06, 3.62 ± 1.02 and 4.14 ± 1.22, respectively, with a significant difference (*P* < 0.05). On the third day after the operation, the VAS values of pain in the three groups were 2.20 ± 0.89,2.62 ± 0.90 and 3.17 ± 0.98, respectively, with significant differences (*P* < 0.05). Postoperative pain was significantly relieved and stabilized. The VAS value of postoperative pain in the three groups was compared, and the postoperative pain in the EAG was significantly better than that in the EBG and CG (*P* < 0.05). See Table 4.

**Table 4:** Comparison of postoperative pain VAS values among the three groups of patients (*n* = 200, $\overline{x}$ ± s)

Group	Number of cases	1d after surgery	2d after surgery	3d after surgery
EAG	84	3.89 ± 0.96	3.05 ± 1.06	2.20 ± 0.89
EBG	53	4.19 ± 1.29	3.62 ± 1.02	2.62 ± 0.90
CG	63	4.92 ± 1.05	4.14 ± 1.22	3.17 ± 0.98
χ [2] /F		16.58	17.90	20.06
*P*		0.000	0.000	0.000

### Comparison of swelling grade scores on the first day after surgery among the three groups

On the first day after the operation, the proportion of patients with swelling grades 0 and 1 in the EAG accounted for 72.6%, and the proportion of patients with swelling grades 2 and 3 was only 27.4%. The swelling grade of EBG was 60.3%, and the proportion of 2 and 3 points was only 39.7%. The swelling grade of CG was 46%, and the proportion of 2 and 3 points was only 54%. The facial swelling of the experimental EAG was better than that of the EBG and CG (*P* < 0.05). See Table 5.

**Table 5:** Comparison of swelling grade scores on the first postoperative day in the three groups (*n* = 200, cases)

Group	Number of cases	0分	1分	2分	3分
EAG	84	17	44	20	3
EBG	53	4	28	17	4
CG	63	0	29	26	8
χ^2^	22.50				
*P*	0.001				

### Comparison of the three groups of patients with limited mouth opening grade scores on the first day after surgery

On the first day after the operation, 75.0% of EAG had a limitation of 0 and 1 points, and only 25.0% had 2 and 3 points. In the EBG, the proportion of 0 and 1 points was 70.4%, and the proportion of 2 and 3 points was only 39.6%. In the CG, the proportion of 0 and 1 points was 47.6%, and the proportion of 2 and 3 points was only 52.4%. The limitation of mouth opening in the EAG was better than that in the EBG and CG (*P* < 0.05). See Table 6.

**Table 6:** Three groups of patients after the first day of the mouth opening limitation grade score comparison (*n* = 200, cases)

Group	Number of cases	0分	1分	2分	3分
EAG	84	22	41	16	5
EBG	53	8	24	15	6
CG	63	4	26	24	9
χ^2^	16.33				
*P*	0.012				

### Comparison of postoperative complications among the three groups

The incidence of postoperative complications in the three groups was 7.1, 17.0, and 36.5%, respectively. The EAG was lower than the EBG and CG (χ2 = 21.69, *P* = 0.017, *P* < 0.05). See Table 7.

**Table 7:** Comparison of postoperative complications among the three groups (n = 200, cases)

Group	Number of cases	WI	DSD	FB	JD	LLN	Total
EAG	84	2	1	1	0	2	6(7.1%)
EBG	53	2	1	3	1	2	9(17.0%)
CG	63	7	3	5	3	5	23(36.5%)
χ^2^	21.69						
*P*	0.017						

All three of the facial bruises in the EBG were subcutaneous emphysema, while none of the cases in the EAG and CG were present.

## Discussion

Extraction of mandibular medium- and low-impacted wisdom teeth is a common procedure in dentistry, which has problems such as difficult extraction, significant postoperative pain, and more adverse reactions, making it a more difficult type of dental extraction. Before extraction, patients should improve the relevant imaging examinations (CBCT if necessary), comprehensively assess the possible risks and difficulties during extraction, and develop a scientific and effective surgical treatment method to effectively improve the treatment results.

For operation time, in the study of Rossi et al. [16], the UBK operative time was longer than that of high-speed turbine handpieces because they cut bone and dental tissues more slowly. Other scholars believe that compared with UBK, the high-speed turbine handpiece can quickly cut bone tissue and tooth tissue, greatly reducing the operation time. However, when the high-speed turbine handpiece cuts bone tissue, the working tip is heated, causing the surrounding temperature to rise and causing thermal damage to bone tissue.

In terms of surgical bleeding, in the study of Rude et al. [17], their conclusions were that the advantages of precise identification and selective cutting of the ultrasonic bone tool can greatly reduce the amount of surgical bleeding while also effectively reducing postoperative exudation. The reason is that the cavitation effect of normal saline will form a bubble in the body and implode into the blood vessel, resulting in a shock wave, resulting in micro-condensation in the blood vessel, which plays a hemostatic role [18].

**Fig. 20:**
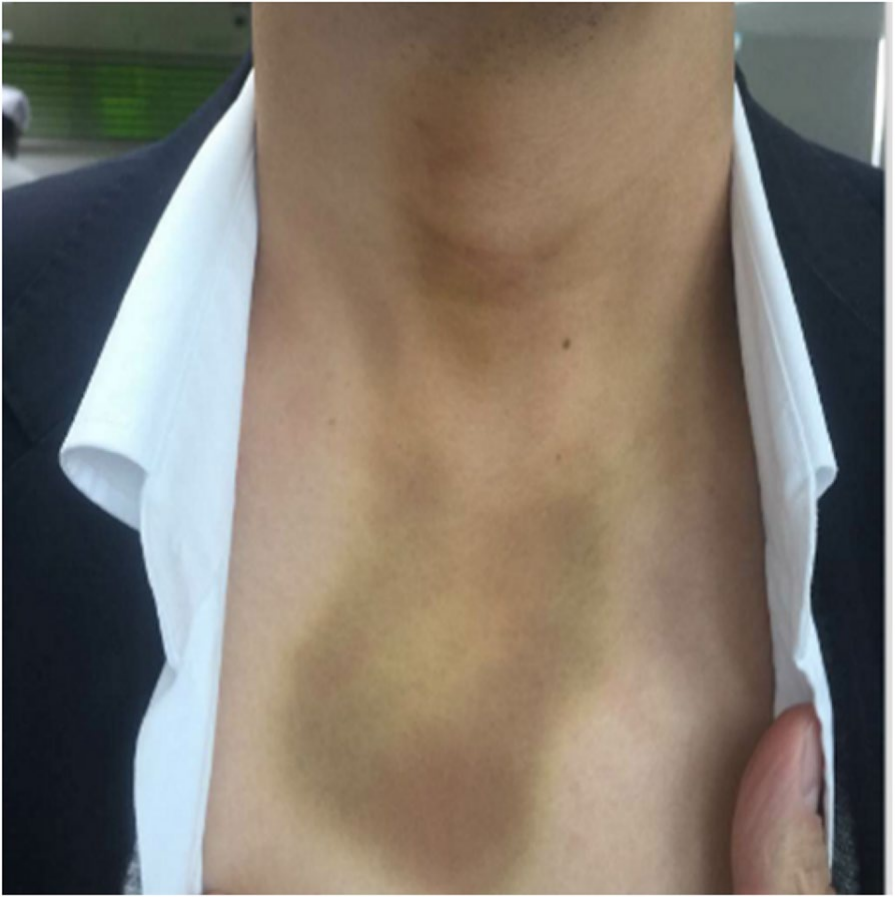
Subcutaneous emphysema

**Fig. 21:**
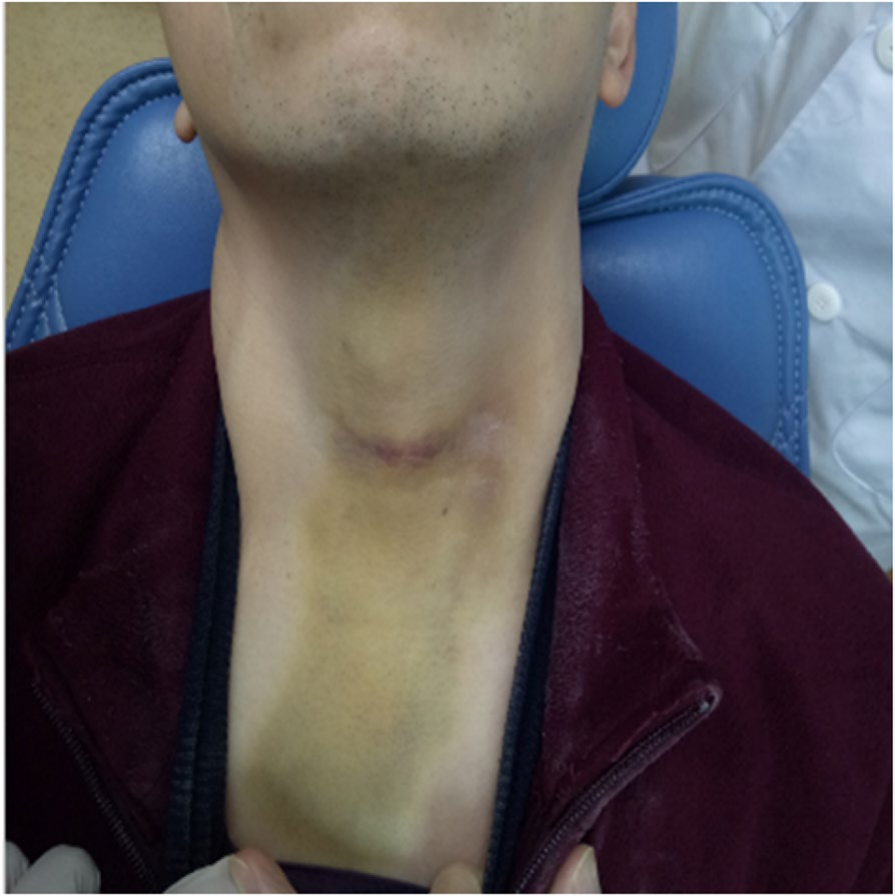
Subcutaneous emphysema

**Fig. 22:**
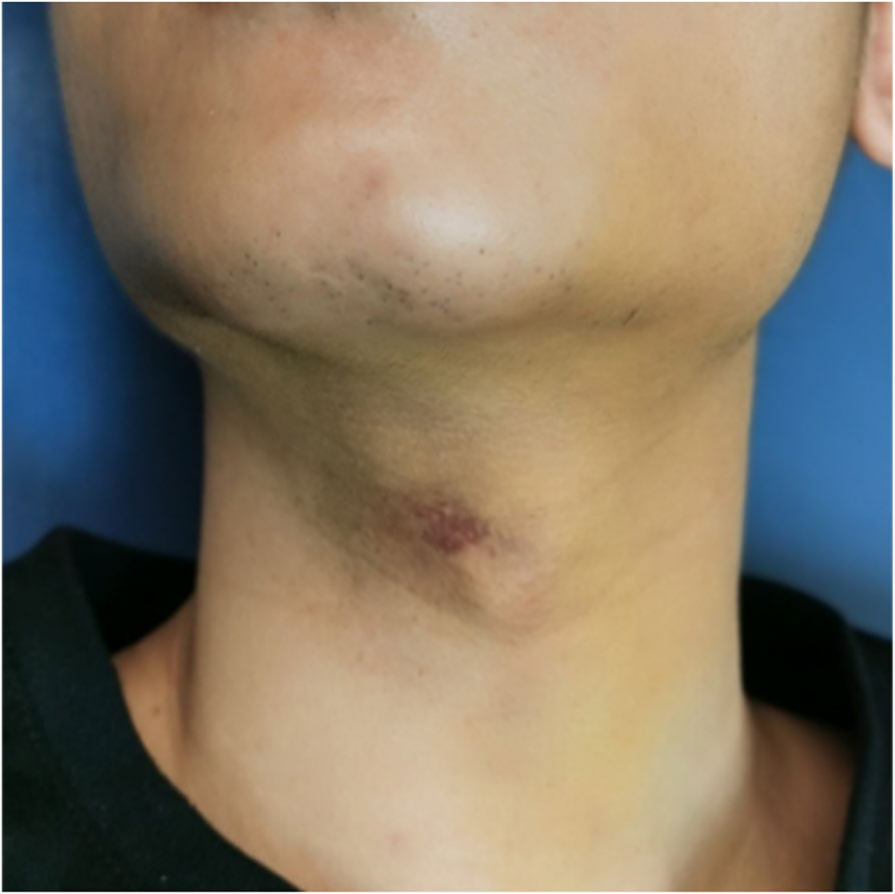
Subcutaneous emphysema

In terms of postoperative complications, Bassi et al. [19] concluded that UBK can reduce postoperative pain, swelling, and limited mouth opening compared to high-speed turbine handpieces and bone chisel splitting. For the incidence of postoperative complications, the study of Jenkins et al. [20] confirmed that ultrasonic osteotome had a significantly lower complication rate for postoperative occurrence of dry socket, inferior alveolar injury, and subcutaneous emphysema (see Figs. 20, 21 and 22).

The application of the UBK treatment method, which is a new type of minimally invasive extraction technique, converts electrical energy into mechanical energy when using microcrystalline and ceramic technology to generate charge flow action, and through ultrasonic shock is the vaporization of water in cells and tissues and the breaking of hydrogen bonds, resulting in the destruction of the bone it contacts [21]. In the process of cutting, important anatomical structures such as soft and hard tissues, nerves and blood vessels can be distinguished, and the cutting range can be accurately controlled. Accuracy control is very accurate, which can effectively improve the rationality of surgical cutting [22]; and has an adjustable cutting intensity mode to avoid serious damage to the mucosa and vascular nerves, which can minimize the risk of soft tissue damage during the cutting process, avoid the blindness of traditional treatment methods, reduce intraoperative bleeding, and facilitate a clear visual field; it can effectively reduce postoperative complications such as numbness and dry groove syndrome incidence, and the treatment is safer and more precise [23].

UBK uses different frequencies to cut soft and hard tissues. During the cutting process, it causes less damage to the surrounding tissues and promotes bone tissue healing and repair regeneration. The released oxygen ions have a certain anti-corrosion effect and will not cause osteonecrosis or even osteonecrosis near the cutting area. When the ultrasonic bone cutter osteotomy forms an irregular rough surface, and on this rough bone edge alternate formation of osteoclasts and osteoblasts, osteoblasts are larger than osteoclasts to promote bone growth. However, the turbine handpiece has certain limitations: first, the jet of air causes gas to enter the tissue, easily causing subcutaneous emphysema; second, the higher heat production of the high-speed turbine handpiece causes excessive temperature, which leads to marginal osteonecrosis and may affect postoperative bone regeneration and bone healing; and third, the waterway system is not effectively sterilized, which may cause infection [24].

The new DEM has a high ability to cut dental tissues, reducing the operative time and postoperative complications such as subcutaneous emphysema; the DEM has a higher cutting efficiency and better stability in both debridement and crown division. Because of its excellent performance, such as providing auxiliary light illumination, good cooling effect, high speed and efficiency, it will not stop working when the pressure is too high. In addition, UBK does not cause bone tissue necrosis due to its characteristics and advantages, such as precise cutting, ability to stop bleeding, clear field of view, no high-speed rotation, no scraping of the surrounding tissue, and easy control [6]. Due to the low cutting efficiency of UBK, it is often used in combination with DEM, which improves the efficiency of debridement and cutting dental tissues to some extent, thus reducing the operation time.

In this study, three groups of patients were given different surgical treatment methods, namely, the chiseling and splitting crown method, ultrasonic osteotome method combined with DEM and turbine mobile phone. The chiseling and splitting crown method is a more traditional extraction method in clinical practice. It mainly uses continuous knocking force to remove the cortical bone and teeth around the impacted wisdom teeth. The knocking force during treatment will not only cause fear and tension in patients., resulting in a decrease in patient compliance but also, cause other periodontal soft tissues to suffer from certain trauma and serious postoperative complications. Therefore, it requires higher proficiency of the operator. Traditional treatment methods have certain limitations, so the majority of scholars and clinicians have turned their attention to “minimally invasive” technology. Many studies have shown that the clinical effect and safety of UBK in the extraction of mandibular middle and low impacted wisdom teeth are better than those of chiseling and crown splitting [25, 26].

This experiment shows that the operation time of UBK combined with DEM is relatively shorter. The author analyses that it may be related to the following reasons: the “cavitation effect” produced by the UBK causes the water mist around the working end to produce a uniform shock wave, which limits the blood exudation, clears the debris tissue in the operation area, and facilitates the operation of the surgeon [27]. The experimental group maintained the activity of wound tissue to the greatest extent, which was helpful to wound healing, reduced the impact and trauma on the lower collar joint, and reduced the degree of postoperative mouth opening limitation and pain. In addition, minimally invasive technology causes less damage to patients, a small surgical incision and minimally invasive characteristics, so it can effectively reduce the anxiety of patients and facilitate their acceptance.

The data of this clinical study confirmed that compared with the control group, patients in A and B had a shorter operation time, better psychological state during operation, lower pain VAS value on the 1st, 2nd and 3rd day after operation, lower degree of facial swelling and mouth opening restriction on the 1st day after operation, and lower incidence of postoperative complications (*P* < 0.05). Compared with the traditional wisdom tooth extraction method, UBK combined with the DEM method can take into account the efficacy and safety of treatment, which has significant advantages and high clinical application value. Compared with HSTMP, the new DEM is more efficient, safer, has less noise and fewer postoperative complications and is widely favored by surgeons.

In summary, the application of UBK combined with DEM in the extraction of mandibular middle and low impacted wisdom teeth has good application and good effect. It can effectively improve the quality of life of patients after operation, reduce intraoperative blood loss and operation time, and greatly reduce the subcutaneous emphysema caused by the jet airflow into the tissue of TSTMP and the fear and complications after traditional extraction. It is worthy of wide application.

## Conclusion

The use of a UBK combined with a DEM was used to extract mesial, low-level or proximal mesial obstruction of the mandibular third molar near the alveolar nerve. This procedure can reduce the operative time and intraoperative bleeding, postoperative pain, facial swelling, and mouth opening restriction, improve patient comfort, and reduce the incidence of postoperative complications, which greatly ensures patient safety. Therefore, it is recommended that the surgical approach of UBK combined with DEM be considered first for the extraction of obstructed teeth near the alveolar nerve canal. However, the application of UBK combined with HSTMP was first considered to have low work efficiency during tooth division, and high heat production may cause complications such as bone tissue necrosis and subcutaneous emphysema.

### Supplementary Information

**Additional file 1.**

**Additional file 2.**

**Additional file 3.**

**Additional file 4.**

## Data Availability

The datasets generated and/or analyzed during the current study are not publicly available due to privacy and ethical concerns but are available from the corresponding author upon reasonable request.

## Abbreviations

UBK: Ultrasonic bone knife
DEM: Dental electric motor
HSTMP: High-speed turbine mobile phone
BCSC: Bone chisel split crown
EAG: Experimental group A
EBG: Experimental group B
CG: Control group
CBCT: Cone beam computed tomography
VAS: Visual analog scale
LSD: Least significant difference
WI: Wound infection
DSD: Dry socket disease
FB: Facial bruising
JD: Joint discomfort
LLN: Lower lip numbness
