# Supplementary figures and images for: Application of an ultrasonic bone knife combined with a dental electric motor in the extraction of mandibular middle and low impacted teeth
Source: BMC Oral Health. 2024 Jan 4;24:18. doi: 10.1186/s12903-023-03788-0 (PMC10768189; doi:10.1186/s12903-023-03788-0)

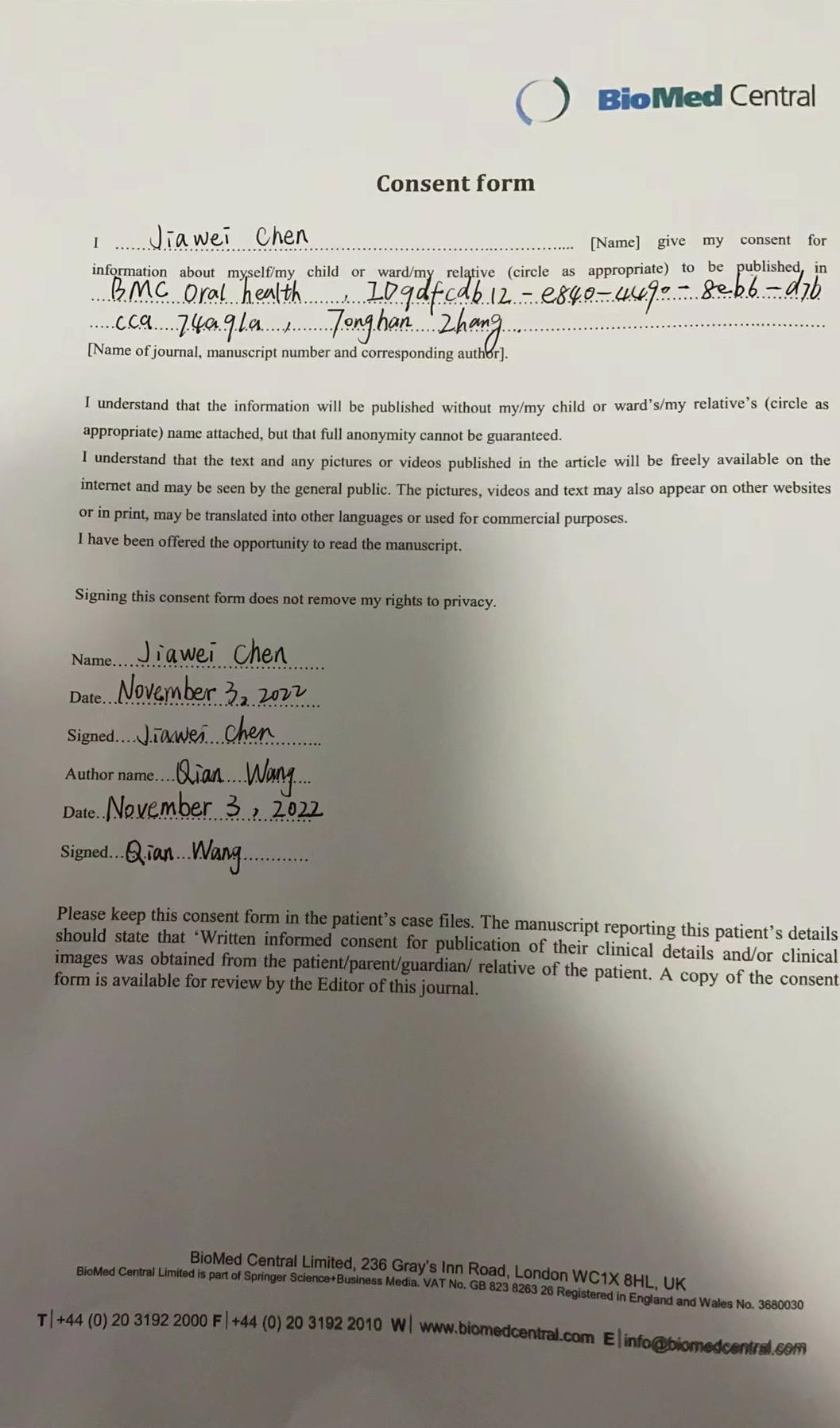

Supplement: Supplementary file 1 — Additional file 1. [file 12903_2023_3788_MOESM1_ESM.jpg]

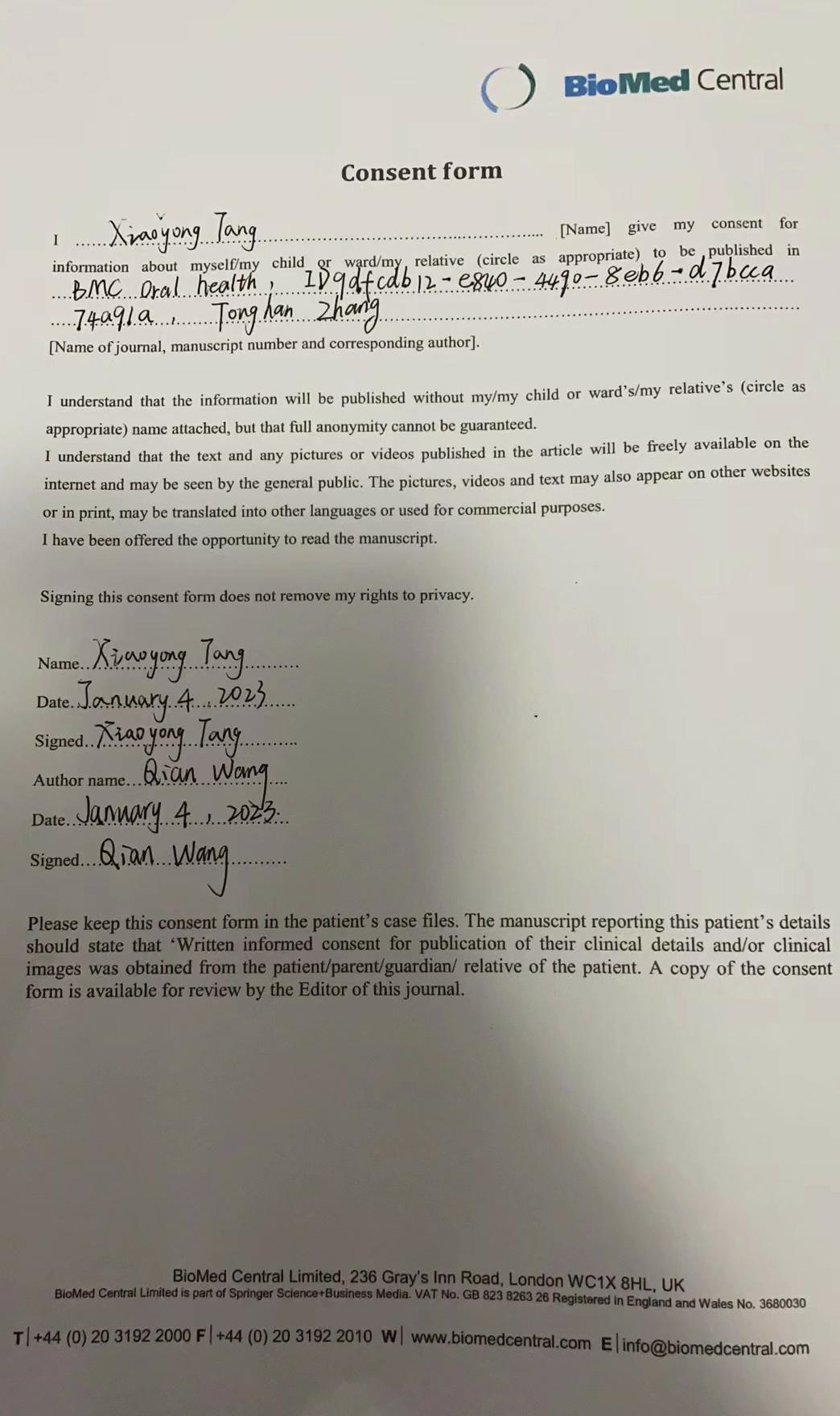

Supplement: Supplementary file 2 — Additional file 2. [file 12903_2023_3788_MOESM2_ESM.jpg]

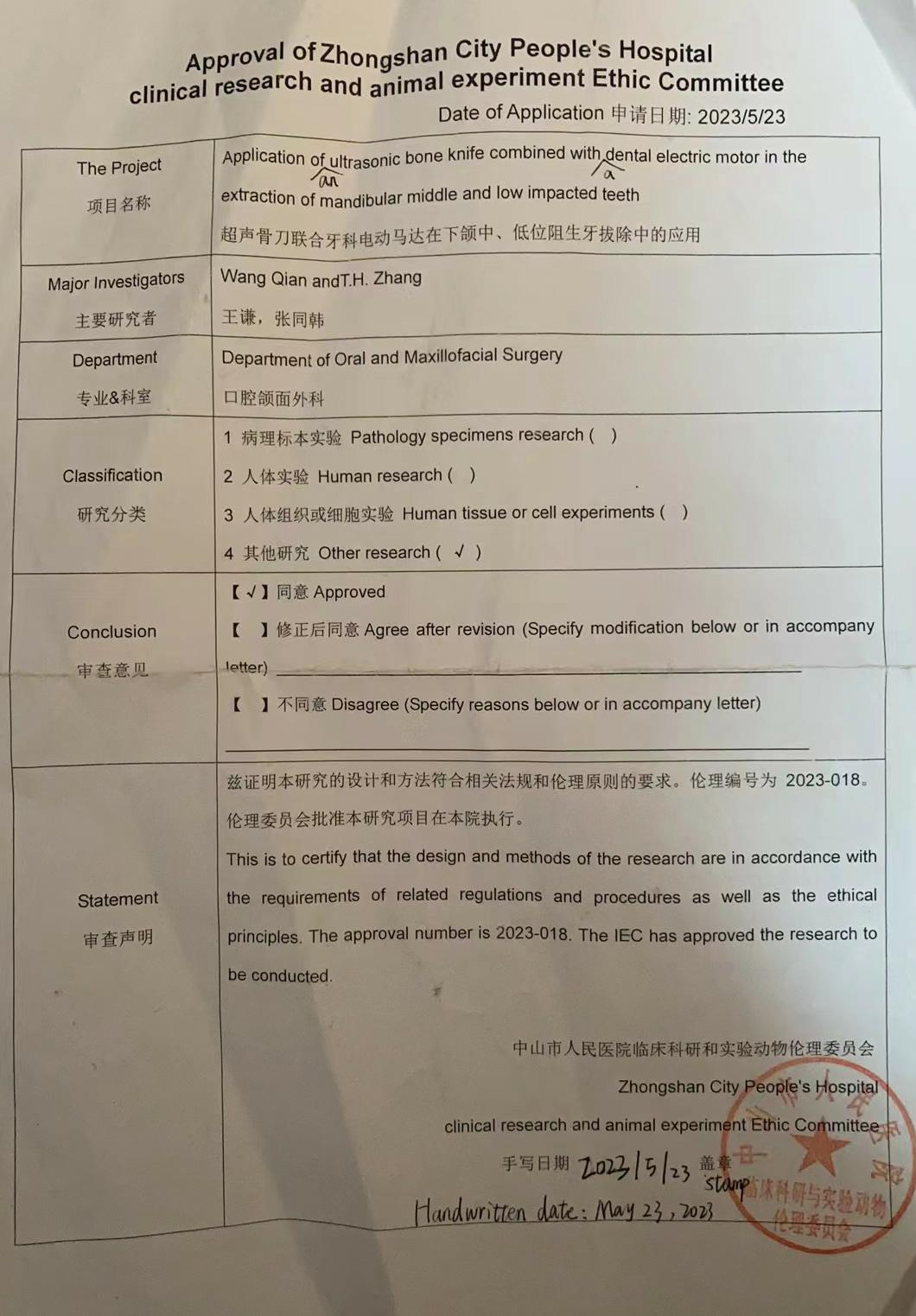

Supplement: Supplementary file 3 — Additional file 3. [file 12903_2023_3788_MOESM3_ESM.jpg]
